# Regulation of the Migration of Distinct Dendritic Cell Subsets

**DOI:** 10.3389/fcell.2021.635221

**Published:** 2021-02-19

**Authors:** Meng Feng, Shuping Zhou, Yong Yu, Qinghong Su, Xiaofan Li, Wei Lin

**Affiliations:** Institute of Basic Medicine, Shandong First Medical University & Shandong Academy of Medical Science, Shandong Provincial Hospital Affiliated to Shandong First Medical University, Jinan, China

**Keywords:** dendritic cells, migration, conventional dendritic cells, plasmacytoid dendritic cells, chemokines, adhesion molecules

## Abstract

Dendritic cells (DCs), a class of antigen-presenting cells, are widely present in tissues and apparatuses of the body, and their ability to migrate is key for the initiation of immune activation and tolerogenic immune responses. The importance of DCs migration for their differentiation, phenotypic states, and immunologic functions has attracted widespread attention. In this review, we discussed and compared the chemokines, membrane molecules, and migration patterns of conventional DCs, plasmocytoid DCs, and recently proposed DC subgroups. We also review the promoters and inhibitors that affect DCs migration, including the hypoxia microenvironment, tumor microenvironment, inflammatory factors, and pathogenic microorganisms. Further understanding of the migration mechanisms and regulatory factors of DC subgroups provides new insights for the treatment of diseases, such as infection, tumors, and vaccine preparation.

## Introduction

Dendritic cells (DCs) are professional antigen-presenting cells that link innate and adaptive immune responses. In 1973, scientists isolated cells with unique dendritic processes from the peripheral lymphoid organs of mice and named them “dendritic cells” (Steinman and Cohn, 1973). Subsequently, Idoyaga and Steinman (2011) found that DCs participated in adaptive immune response after continuous migration and activation. The function of DCs, whether in maintaining immune tolerance or promoting immunity, require migration to a certain target destination. Recent studying has brought new ideas into the development of different DC subsets in immune responses. Herein, we reviewed the DC subsets that have been reported in recent years and discussed the regulatory factors and molecular mechanisms involved in DC migration. Elucidating the mechanisms underlying the migratory DCs would contribute to the development and function of different DC subsets and their role in diseases.

### DC SUBSETS

**FIGURE 1:**
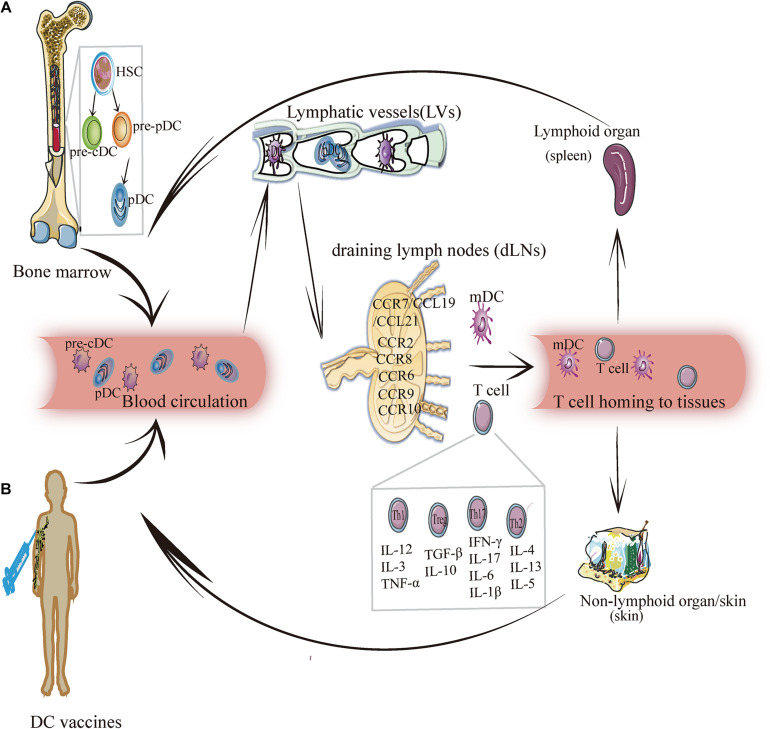
The migration of DC subsets. **(A)** DC endogenous migration: The precursor DC (pre-DC) develops from hematopoietic stem cells and gradually differentiates into pre-cDC and pDC. Then pDC and pre-cDC migrates from the bone marrow and enters blood circulation (in this case, DC is imDC and pre-cDC differentiates into cDC). Under the mediation of chemokines and cytokines, imDC enters lymphatic vessels, and then reaches draining lymph nodes. In this process, imDC relies upon chemokines (for example: CCR7/CCL19/CCL21, CCR8, CCR6, CCR9, CCR10, etc.) to migrate and transform into mDCs and induce T cells (Th1, Th2, Th17, and Treg) to migrate into lymphoid tissues (such as: the spleen) or non-lymphoid tissues (such as: skin) to exert an immunity effect. **(B)** DC exogenous migration: After the human body is injected with the DC vaccine, DCs loaded with specific antigens enter the blood circulation, and crawls along the blood vessel wall to reach the lymphatic vessels, and then enters the draining lymph nodes to activate the adaptive immune response, by which it exerts anti-tumorigenic or antiviral effect.

Dendritic cells are highly heterogeneous cells that have historically been categorized by phenotype, function, or location. DCs are unique hematopoietic cells that originate from precursor cells, such as monocytes and pre-DCs, in bone marrow (Naik et al., 2006; Liu et al., 2009; Liu and Nussenzweig, 2010). Precursor cells migrate to peripheral tissues and secondary lymphoid organs via blood circulation and/or lymphatic vessels where they differentiate into myeloid DCs and lymphoid DCs (Naik et al., 2007) (Figure 1). According to specific transcription factors and chemokines, these DCs are further differentiated into three classic subsets: conventional DC1s (cDC1s), conventional DC2s (cDC2s), and plasmacytoid DCs (pDCs). According to their states of maturity, DCs are divided into immature DCs (imDCs), mature DC (mDCs), semi-mature DCs (smDCs), and tolerogenic DCs (tol-DCs). Semi-mature DCs (smDCs), which are an activation state between immature and mature DC cells, are difficult to define (Lutz and Schuler, 2002). These classic DC subsets play a critical role in regulating immune response and immune tolerance.

### cDC1s

Conventional DC1s widely exist in the blood and peripheral tissues of human and mouse, but their expression is very low in mouse blood. Mouse cDC1s have strong homogeneity in expressing CD8 and/or CD103 (Edelson et al., 2010). Mouse CD8^+^ cDC1s are identified as CD11c^*hi*^CD45R^–^MHCII^+^ CD8α^+^DEC205^+^CD11b^*lo*^Sirpα^*lo*^ and express C-type lectin Clec9A (DNGR1), Nectin-like protein 2 (Necl2; also called CADM1). Migratory CD103^+^ DCs in most non-lymphoid tissues are defined as CD11c^+^MHCII^+^CD103^+^CD11b^*lo*^CX3CR1^–^ F4/80^–^Sirpα^–^ (McLellan et al., 2002; Bursch et al., 2007; Huysamen et al., 2008; Ginhoux et al., 2009). Both resident CD8a^+^ and migrating CD103^+^ cDCs express CD36, CD24, and XCR1 and play a critical role in immunity against intracellular pathogens, viruses, and cancer. In mouse blood, activated cDC1s secrete interleukin (IL)-12p70 and induce the T helper type 1 (Th1) response (Maldonado-Lopez et al., 1999; Farrand et al., 2009). Human CD141^+^/BDCA-3^+^ Conventional DC1s are primarily distributed in lymphoid tissues, express C-type lectin receptor 9 (Clec9) and X-C motif chemokine receptor 1 (XCR1), and contribute to antiviral immunity (Silvin et al., 2017), whereas human thymus CD141^+^cDC1 produces high levels of IL-12 and induces the Th17 response (Vandenabeele et al., 2001). A group of specific DC subgroup Langerin^+^(CD207^+^)CD103^+^CD8^+^cDC1 was found in the human spleen, and it was a key regulator of immune responses toward blood-borne antigens in the steady-state and during inflammation (Backer et al., 2019). In bacteria-infected human or mouse skin, a subset of CD59^+^EpCAM^+^Ly6D^+^ cDC1 promotes the infiltration of numerous neutrophils by producing the vascular endothelial growth factor (VEGF)-α (Janela et al., 2019). cDC1 contributes to antigen presentation, induces angiogenesis, and promotes inflammation.

The migration of cDC1 is primarily correlated with CXCR3 and CCR7 expression. CXCR3 expression is restricted to mice pre-cDC1 and pDC lineages and is specifically expressed in pre-cDC1 (Siglec-H^–^Ly6C^–^) but not pre-cDC2 (Siglec-H^–^Ly6C^+^). Trafficking to periphery CCR7-CCL21α^–/–^ interactions guides the migration of pre-cDCs (Lin^–^CD11c^+^MHCII^–^Flt3^+^Sirpα^*lo*^), which accumulate in the thymus, where they may be important for T-cell tolerance (Cosway et al., 2018).

### cDC2s

Conventional DC2s have high heterogeneity and play dual roles of immune activation and regulation in the immune response. In the blood, activated cDC2s secrete IL-1β, IL-6, and IL-23 and induce the Th17 response (Persson et al., 2013). In the mouse intestine and thymus, cDC2s can induce the production of regulatory T cells (Treg) (Proietto et al., 2008; Balan et al., 2019). Recently, human cDC2s have been divided into two subsets: (1) CD1c^*lo*^CLEC10A^–^CLEC4^*hi*^ cDC2A expresses a high level of amphiregulin (Areg) and matrix metalloproteinase-9 (MMP-9) but low levels of IL-23, IL-6, and tumor necrosis factor-α (TNF-α). This subset exhibits anti-inflammatory effects. (2) CD1c^+^CLEC10A^+^CLEC4^*lo*^ cDC2B has pro-inflammatory effects with high expression levels of IL-6 and TNF-α (Brown et al., 2019). The corresponding two subgroups of cDC2s in mice are T-bet^+^ cDC2A and T-bet^–^ cDC2B (Brown et al., 2019), which are different from the previously described cDC2 subsets. Notch2 targeting of CD11c^+^CD11b^+^ CD103^+^ IRF4^+^ cDC2s was associated with the induction of the Th17 cell response (Lewis et al., 2011), whereas Kruppel-like factor 4 (Klf4)-dependent CD11c^+^ IRF4^+^ cDC2s promote Th2, but not Th17 (Tussiwand et al., 2015). In addition, CD9 divided CD11b^+^cDC2s into two subgroups in B16-F10 tumor-bearing mice, namely CD9^–^(CD301^–^)/CD9^+^(CD301^+^)CD11b^+^cDC2s, which are required for activating antitumor CD4^+^ T_*conv*_ (Binnewies et al., 2019). Although cDC2s are divided into many subsets, migratory cDC2s subsets typically require CCR7, whereas extrathymic Sirpα^+^cDC2s enter the thymus primarily via CCR2 (Tomohisa et al., 2009).

### pDCs

Plasmacytoid DCs were first discovered in human lymph nodes (LNs). Human CD11c^–^CD123^+^CD303^+^ pDCs are equivalent to mice PDCA-1^+^ pDCs. pDC differentiation depends on E2.2 and IRF7, and expresses the CD123/IL-3α chain, CD303 (BDCA-2), CD304 (BDCA-4), and immunosuppressive molecule ILT2, etc. (Dzionek et al., 2000; Swiecki et al., 2010; Mathan et al., 2013). A new subgroup, AXL^+^AS DCs (SIGLEC1^+^, SIGLEC6^+^), exists in human blood and expresses a similar marker as that of pDCs (Villani et al., 2017). Although these subgroups are incapable of proliferation, they can activate T cells and play an antiviral role. Furthermore, pDCs can be converted into cDCs. When transcription factor E2-2 is downregulated or ID2, PU.1, and BATF3 are significantly upregulated, CC-chemokine receptor 9 (CCR9)^–^ pDCs in intestinal epithelial cells (IECs) migrate to peripheral tissues (Chen et al., 2015). Subsequently, they are transformed into CD11b^+^CD8^+^MHCII^+^ cDC-like cells under the stimulation of granulocyte-macrophage colony-stimulating factors (GM-CSF) or soluble factors produced by IEC (Schlitzer et al., 2011). This transformation leads to an imbalance or abnormal distribution of pDC and cDC subpopulations in the body, which induce autoimmune diseases (Chen et al., 2015; Qian and Cao, 2018). CCR4, CCR6, CCR7, CCR9, CCR10, and chemokine-like receptor 1/chemerin receptor 23 (CKLR1/ChemR23) are correlated with pDC migration (Penna et al., 2001; Vermi et al., 2005; Wendland et al., 2007; Sisirak et al., 2011).

### tol-DCs

Tolerogenic DCs (tol-DCs) can be derived from monocytes or pre-DCs. GM-CSF and TGF-β1 stimulated mouse liver-derived pre-DCs into tol-DCs, which prolong the survival time of donors in organ transplantation (Bonham et al., 1996; Khanna et al., 2000). These tol-DCs induce Treg cells to exert immune tolerance by secreting large amounts of IL-10. In addition, skin-settled CD141^+^CD14^+^ DC are derived from colonized monocytes (Chu et al., 2012; Han et al., 2014), and inhibit the CD4^+^ T-cell response by secreting IL-10 and IDO. IDO^+^CD11b^+^ DC is a subset of tol-DCs (Park et al., 2012) and induces immune tolerance. Tol-DCs are classified as induced tolerogenic DCs (itDCs) and natural tolerogenic DCs (ntDCs). ItDCs contribute to the maintenance of homeostasis under potentially proinflammatory conditions. While under steady-state conditions, ntDCs facilitate the establishment of tolerance. These findings provide insights on a new framework for the use of DC-mediated mechanisms of tolerance to treat diseases (Iberg and Hawiger, 2020).

## Mechanism Underlying DC Migration

Migration is the key process through which DCs exercise their uptake, processing, and presentation, and it runs throughout the entire process of DC differentiation and development. DC migration affects its phenotype and maturity, thus resulting in the different localization of different DC subgroups. DCs can directly pass through the blood vessel wall and migrate from peripheral tissues to a specific location or can enter lymph vessels from the bloodstream, from which they are passively transported to the subcapsular sinus (SCS) of the LNs through lymph flow and enter LNs to complete migration (Figure 1A).

### Migration Kinetics of DCs

Differentiation and development of DCs occurs in four stages: (1) DC precursors, (2) imDCs, (3) migration DCs, and (4) mDCs. Previous studies have shown that only CD34^+^ DC precursor cells express E-cadherin, which promotes DC migration and maturity (Mackensen et al., 2000). During acute inflammation, DC precursors quickly mobilize to non-lymphoid tissues. Most DCs in the peripheral organs are imDCs, as immune response sentinel, which can take up antigens more efficiently. With exogenous antigens and inflammatory factor stimulation, imDCs migrate from peripheral tissues to secondary lymphoid areas. During this process, imDCs develop into mDCs, which present antigens and induce the T-cell response in LNs (Caux et al., 2000). Differentiation from imDCs to mDCs depends on migratory DCs. This type of DC exists mainly in lymphatic tissues, input lymphatic vessels, and peripheral blood. Through blood and lymphatic circulation, migratory DCs enter the secondary lymphatic organs from the input lymphatic vessels and drive the DCs to mature.

The dynamic migration process of DCs has an important guiding role in elucidating their homeostasis and pathology in tissues. However, different DC subsets display distinct migration kinetics during migration from skin to the draining LN (dLN). After photoconversion, self-antigens that are present on CD103^–^ dermal DCs are rapidly transported from the skin to the dLN and are responsible for the transport of invading pathogens to the dLN. In contrast, CD103^+^ DCs reached a plateau on day 3 after photoconversion and participated in antigen cross-presentation (Tomura et al., 2014). Moreover, different DC subsets survey different regions of the spleen to induce specific T cell responses. For example, 33D1^+^ DC migrates to the periphery of the T cell zone of the spleen to induce CD4^+^ T cell responses, whereas XCR1^+^ DC migrates to the center of the T cell zone in the white pulp of spleen to induce CD8^+^ T cell responses (Calabro et al., 2016). In addition, the migration of localized skin CD1c^+^/CD14^+^/CD141^+^ DC subgroups to the inflammation site depends on CCR7/CXCL10 (Chu et al., 2012; Haniffa et al., 2012, 2015). However, research on the migration of a certain DC subgroup to a specific site in the tissues under steady-state and inflammatory conditions remain insufficient. Furthermore, whether independent DC subsets can selectively induce the T cell response in other immune organs warrants further research.

### Essence and Mechanism of Migration

The migration of DCs is a complex and dynamic cyclical process. Cell migration occurs due to interaction between chemokines and chemokine receptors. Under the guidance of chemokines, DCs move to specific sites and exert corresponding biological functions. In addition, adhesion factors, integrins, semaphores, and cytoskeletal proteins play various roles in cell migration. Furthermore, the migration of DCs is essential for T cell responses.

#### Chemokines

Chemokines and chemokine receptors guide the positioning and chemotaxis effects of DCs at different developmental stages. Chemokines are a class of highly conserved small, secreted proteins that regulate DC migration by identifying chemokine receptors that bind to the DC surface. CCR7 plays an important role in DC migration from peripheral tissues to draining LNs and is a key factor that affects DC migration and function (Yanagihara et al., 1998; Hirao et al., 2000; Randolph et al., 2004). CCR7 ligands CCL19 and CCL21, which are expressed in lymphoid organs mainly, drive DC migration (Elke et al., 2004; Tiberio et al., 2018). CCL21 forms the “CCL21 gradient” by binding to heparan sulfates in the interstitium, thereby providing adhesion for DC migration and guiding DC migration into LNs (Weber et al., 2013). The discovery of chemokines and the chemokine–chemokine receptor axes facilitates research to elucidate the migration mechanism of DCs.

(1) The CCR7-CCL21/CCL19 axis: The CCR7-CCL19/CCL21 chemokine axis is vital for the regulation of adaptive immunity and tolerance by affecting mDC migration from the peripheral tissue to lymphatic vessels and LNs (Forster et al., 2008). Depending on this axis, human skin CD141^+^CD1c^–^XCR1^+^ cDC1 (Igyártó et al., 2011)/CD1a^+^CD1c^+^cDC2 (Kitajima and Ziegler, 2013) migrates from the dermis to skin-draining LNs (Tamoutounour et al., 2013) or intestinal CD103^+^ CD11b^–^XCR1^+^ SIRPα^–^CD141^+^DNGR1^+^cDC1 (Olga et al., 2009)/ CD103^+^ CD11b^+^ XCR1^–^ SIRPα^+^ CD141^–^DNGR^–^cDC2 (Persson et al., 2013) migrate from the lamina propria to mesenteric LNs (Farache et al., 2013). CCR7-CCL19/CCL21 promote the migration of corneal mDCs to intraocular lymphatic vessels and mediate the CD4^+^ T cell immune response (Wang et al., 2019). Migrations of Newcastle disease virus-like particles (NDV-VLP)-treated DCs to draining LNs or the spleen rely upon the CCR7-CCL19/CCL21 axis, thus leading to CD4^+^ T cell activation (Qu et al., 2005). CCR7-CCL19/CCL21-dependent DC migration is involved in the coordination of the activation of specific Tregs, which is beneficial for maintaining peripheral tolerance (Leventhal et al., 2016). This provides new insights for further understanding the role of the CCR7-CCL19/CCL21 axis in maintaining a balance between the adaptive immune response and immune tolerance. Inflammatory factors CCRL1 (called ACKR4) (Ulvmar et al., 2014), transcription factor PU.1 (Yashiro et al., 2019), and IL-18-driven human helper NK cells (Wong et al., 2014) participated in the regulation of DC migration, which may contribute to adaptive immune responses that are associated with infection, cancer, or vaccination.
(2) The leukotriene B4 (LTB4)-BLT1 axis: This axis is critical for regulating DC transport and inducing an adaptive immune response (Del Prete et al., 2006). DCs can be stimulated by LTB4 *in vitro* and upregulate the expression of CCR7 and CCL19 while promoting chemokines CCL19 and CCL21 to induce DC migration to LNs (Del Prete et al., 2006). This indicates that LTB4 plays an important role in regulating DC migration and inducing adaptive immune responses.
(3) The CXCR4-CXCL12 axis: This axis relies on CCR7 to promote the migration of DCs from peripheral organs to LNs and participate in the migration of DCs across lymphatic endothelial cells and lymphatic vessels, as well as the migration of epidermal DCs to the dermis (Kabashima et al., 2007; Villablanca and Mora, 2008). Thus, CXCR4-CXCL12 is a key axis for DC migration during skin inflammation.
(4) The CCR8-CCL21/CCL8 axis: CCR8 and its ligand CCL21/CCL8 promote DC homing toward LNs (Sokol et al., 2018). In addition, CCR8 and CCL21 coordinate the promotion of CCR7-mediated CD301b^+^ DC migration from the SCS to LNs and induce Th2 effects. Th2 immunization specifically induces CCL8 expression by CD169^+^SIGN-R1^+^ macrophages. CCL8, and CCL21 synergistically promote CD301b^+^ DC migration (Sokol et al., 2018). These factors may contribute to adaptive immune deviation and cancer cell metastasis associated with DC migration.

Some chemotactic signals can directly activate DC migration or promote the production of chemokines (CXCL12, CXCL14, CCL19, CCL3, etc.), thereby causing secondary recruitment of cells (Majumdar et al., 2014; Tiberio et al., 2018). New paradigms have emerged in the establishment and maintenance of gradients during directed cell migration. Such chemotactic signals include bacterial components, lipid mediators, signaling proteins, and proinflammatory cytokines. For example, cathelin-related antimicrobial peptide (CRAMP), platelet-activating factor (PAF), Activin A, serum amyloid A (SAA), and leukotriene B4 (LTB4). The formylpeptide receptor (Fpr2) expressed on the surface of DCs and CRAMP is jointly involved in the activation and aggregation of DCs involved in allergic airway inflammation (Chen et al., 2014). SAA can directly induce the migration of imDCs via the secondary release of CXCL12 and CXCL14 (Gouwy et al., 2015). Furthermore, the chemokine signals induce faster migration of DCs.

#### Adhesion Molecules and Proteins

The acquisition of DC migration capacity also depends on the change of its adhesion state. During inflammation, ICAM-1, ICAM-2, Mac-1 (αMβ2), and LFA-1 (αLβ2) play crucial roles in regulating DC migration. The expression of intracellular chemokine CXCR3 promote Mac-1 and LFA-1 binding to their ligand ICAM-1/2, thereby targeting cell adhesion (Springer, 1994). L/E/P-selectin on activated endothelial cells is required for the DC migration process and is involved in the DC homing of lymphoid and peripheral tissues (Tedder et al., 1995; Pendl et al., 2002; Lekakis et al., 2010).

Tetraspanins are expressed on the surface of DCs and control DC migration by coordinating the expression and aggregation of cytokines, selectins, integrins, or other cell–cell proteins on the DC surface (Charrin et al., 2009). P-selectin-independent rolling decreases in the absence of tetraspanin CD63 (Doyle et al., 2011). Tetraspanin CD53 stabilizes L-selectin surface expression and promotes lymphocyte recirculation (Demaria et al., 2020), which indicates that tetraspanin pairs with its partner protein of selectin in co-participation in DC migration.

Rho-associated protein kinases (ROCKs) affect the migration of DCs to draining LNs by mediating the activation of actin nuclear contraction (Nitschké et al., 2012), rapid reconstruction of F-actin throughout DC migration, cell polarity formation, and interaction between cell proteins (Tang and Gerlach, 2017). The actin-related protein 2/3 (Arp2/3) complex mediated F-actin formation of pseudopodia at the front end of this movement and boosted the CCR7-CCL19/CCL21 response axis to induce chemotactic migration of mDCs (Leithner et al., 2016). Under the regulation of the Rho-GTPase signaling pathway, Arp2/3-mediated actin nucleation weakened at the front end of the migration movement (Suraneni et al., 2015), whereas morphogenetic formin-protein-mediated actin nucleation increased at the end of the migration movement (Vargas et al., 2016), which resulted in the rapid migration of mDCs. Calcium ions maintain cell polarity and stabilize the actin cytoskeleton. DC migration requires the participation of a variety of adhesion factors or proteins, which provides a more comprehensive explanation of the mechanism underlying DC migration.

### Migration Patterns of Different Cell Subsets

**FIGURE 2:**
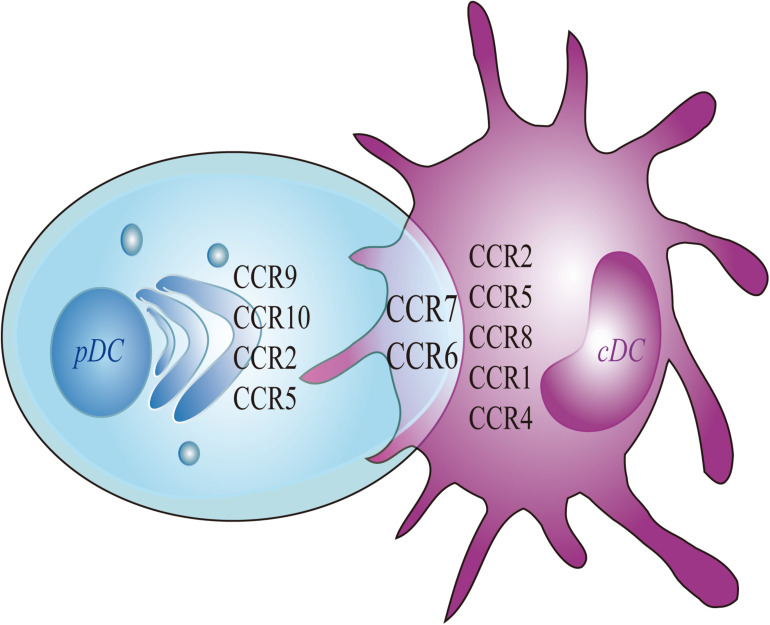
Chemokines of pDC and cDC. Migratory pDC and cDC depends on the expression of CCR7 and CCR6. More specifically, the migration of cDC depends on the expression of CCR8, whereas the migration of pDC depends on the expressions of CCR9 and CCR10. The current study found that the migration of mouse cDC depends on CCR1 and CCR4. The migration of human cDC is dependent on CCR2 and CCR5. At the same time, the expressions of CCR2 and CCR5 are also involved in mediating the migration of mouse pDC.

Migrating cDCs and pDCs recruited from the blood to LNs can promote peripheral Treg cells to induce immune tolerance, thereby linking migrating DCs as potential markers for the treatment of autoimmune diseases (Bonasio et al., 2006; Hadeiba et al., 2012). The development of cDCs and pDCs depends on the expression of CCR6/7 but relies upon CCR1/4/8 or CCR2/9/10, respectively (Figure 2).

Although cDCs usually upregulate the expression of CCR7 to induce migration, during inflammation, IRF4^–/–^CD11b^+^ cDCs could not upregulate CCR7 expression to induce migration to inflamed skin (Bajana et al., 2012; Plantinga et al., 2013). When *Staphylococcus epidermidis* infects skin tissue, dermal CD103^+^ cDC1s carry bacterial antigens that migrate to skin LNs, promote IL-17 secretion, and induce the recruitment of CD8^+^ T cells to the skin to resist pathogen infection (Farache et al., 2013). The chemotactic receptor Epstein–Barr virus-induced 2 (EBI2) can guide the migration of CD11b^+^ cDC2 to the LNs and spleen by up-regulating CCR7, CXCR5, and CXCL13 and inducing CD4^+^ T-cell effects (Gatto et al., 2011; Leon et al., 2012; Gatto et al., 2013). pDCs usually enter LNs through high endothelial veins and assist other DC subsets in performing antigen presentation functions. Upon viral infection, pDCs were directed toward two different sites in the LN, they either migrated to infected macrophages residing in the SCS area in a CXCR3-dependent manner or to CD8^+^ T cell priming sites in a CCR5-dependent manner. This may be essential to induce antiviral immunity (Brewitz et al., 2017). Moreover, CCR9 mediates pDC migration to the intestine (Wendland et al., 2007). CKLR1/ChemR23 mediates pDC migration to LNs or inflamed skin (Zabel et al., 2005; Albanesi et al., 2009). pDCs also depend on CCR6, CCR7, and CCR10 to complete homing from the blood to inflamed skin (Sisirak et al., 2011). pDCs and cDCs express different chemokine and chemokine receptors which lead them to have different migrate route and functions (Table 1). However, the specific mechanism needs to be further studied.

**TABLE 1:** Migration routes, chemokines/chemokines receptor, and functions of cDC and pDC subsets.

	DC subsets	Migration routes	Chemokines/chemokines receptor	Immunological functions	References
cDC1s	Dermis: human CD14^+^CD1a^–^HLA-DR^+^cDC1 (mouse CD11b^+^CD64^+^)	Spontaneously migrate from skin explants cultured *ex vivo*.	CXCL13	Antigen presentation and activation of naive T cells; production of IL-10, IL-6, MCP-1.	Klechevsky et al. (2008); Chu et al. (2012)
	Dermis: human CD141^+^CD1c^–^XCR1^+^cDC1 (mouse CD103^+^CD207^+^/CD8^+^XCR1^+^)	From the dermis to the skin draining lymph nodes via afferent lymphatics.	CCR7	Cross-presentation self-antigen; induction of CD8^+^ effector T cells or Th1 cells; production of TNF-α.	den Haan and Bevan (2002); Crozat et al. (2010), Igyártó et al. (2011); Tamoutounour et al. (2013)
	Intestine: human/mouse CD103^+^CD11b^–^ CD8α^+^ XCR1^+^SIRPα^–^cDC1	From Lamina propria to mesenteric lymph nodes via afferent lymphatics.	CCR7	Cross-presentation self-antigen; induction of CD8^+^ effector T/Th1/Treg cell responses.	Laffont et al. (2010); Cerovic et al. (2015), Esterházy et al. (2016)
	Lung: human/mouse CD103^+^CD11b^–^CD207^+^ XCR1^+^ cDC1	From lung interstitium to mediastinal lymph nodes via afferent lymphatics.	CCR7, CCR2	Cross-presentation self-antigen; induction of CD8^+^ effector T cells; airway tolerance.	del Rio et al. (2007); GeurtsvanKessel et al. (2008), Rose et al. (2010); Fossum et al. (2015), Sharma et al. (2020)
cDC2s	CD11b^+^cDC2	Migrate to the lymph nodes and spleen dependent on EBI2.	CCR7; CXCR5, CXCL13	Cross-presentation self-antigen; induction of CD4^+^ T/Th2 cell responses.	Rose et al. (2010); Gatto et al. (2011), Leon et al. (2012); Gatto et al. (2013)
	Skin: human CD1a^+^CD14^–^ HLA-DR^+^cDC2 (mouse CD11b^+^CD207^–^XCR1^–^)	From the dermis to the skin draining lymph nodes via afferent lymphatics.	CCR7	Antigen presentation and activation of naive T cells or Th2 cell; production of IL-15, IL-8.	Klechevsky et al. (2008); Kitajima and Ziegler (2013), Tamoutounour et al. (2013)
	Intestine: human/mouse CD103^+^CD11b^+^XCR1^–^ SIRPα^+^cDC2	From lamina propria to mesenteric lymph nodes via afferent lymphatics.	CCR7; CCR9/CCL25	Induction of Th1/Th17 cells; induction of Treg cells; production of pro-inflammatory cytokines IL-6, IL-23 and so on.	Farache et al. (2013); Gao et al. (2021)
	Lung: human/mouse CD103^–^CD11b^+^ cDC2	From lung interstitium to mediastinal lymph nodes via afferent lymphatics.	CCR7; CCR1, CCR5	Induction of inflammatory response; induction of protective mucosal immune responses; expression of IL-18, IL-1, or IL-1R.	Lukens et al. (2009); Pang et al. (2013), Sharma et al. (2020)
pDCs	Skin: human CD11c^–^CD123^+^BDCA-2^+^ BDCA-4^+^pDCs (mouse B220^+^ PDCA1^+^ LY6C^+^)	Migrate into inflamed epithelia of mucosae or skin.	CCR6, CCR10	Cross-presentation self-antigen, induction of CD4^+^ effector T cells; production of IFN-γ.	Sisirak et al. (2011)
	Intestine: human CD11c^–^ CD123^+^BDCA-2^+^BDCA-4^+^pDCs (mouse CD11c^*mid*^ B220^+^PDCA1^+^ LY6C^+^)	Homing to the small intestine via high endothelial venules.	CCR9, CCR7	Imbalance of Th1/Th2 effects; production of TNF-α.	Wendland et al. (2007)
	Viral infection sites: pDCs	Migrate into the subcapsular sinus area or CD8^+^ T cell priming site.	CXCR3; CCR5	Induction of CD8^+^ effector T cells; antiviral immunity.	Brewitz et al. (2017)

During inflammation, the migration of imDCs mainly depends on the mediation of E/P-selectin (Pendl et al., 2002). Endothelial selectins are involved in the rolling, extravasation, and migration of imDC in the vascular endothelium. The ChemR23 ligand chemerin can increase the migration of imDCs to endothelial cells with the participation of CCL7 (Gouwy et al., 2013). imDCs and mDCs may have the opposite reactivity to the same chemokine. For example, imDCs have weak reactivity to CC-chemokine-MIP-1b and CXC-chemokine-SDF-1a, but the reverse occurs when imDCs are mature (Lore et al., 1998). In addition, the Rho-mDia1-dependent actin pool is involved in the forward movement of imDCs and the migration of mDCs to lymphatic vessels (Vargas et al., 2016). The migration patterns of different subgroups affect the progress of immune regulation, but the migration mechanism of each subgroup is unclear.

## Regulatory Factors Affecting DC Migration

### The Hypoxic Microenvironment

A sufficient oxygen environment is required to maintain the normal development and metabolism of cells. Hypoxia can downregulate the expression of CCR7 and DC surface adenosine receptor A2b, whereas the cyclic AMP/protein kinase A signaling pathway reduces the inhibition of *MMP-9/TIMP* gene secretion during hypoxia by acting on the A2b receptor (Qu et al., 2005). The downregulation of CCR7 and the change in *MMP-9/TIMP* gene expression are the main factors that inhibit DC migration, which cause an imbalance in the Th1/Th2 immune response.

### Tumor Microenvironment

Elucidating the migration processes of DCs in the tumor microenvironment (TME) can explain how DC-derived cancer vaccines will effectively work in the human body, thus leading to the development of effective vaccines. However, knowledge of the exogenous migration pathway of DC is limited. When tumors occur, increased secretion of TGF-β, VEGF, and LXR ligands and anti-inflammatory factors may recruit DC precursors and convert them into tol-DCs, thereby inhibiting DC maturation and migration to LNs (Soudja et al., 2011). Likely, TGF-β may be involved in DC migration under phosphatidylinositol 3-kinase/Akt activation (Bakin et al., 2000) and increase cell tolerance (Lee et al., 1998). This confirms that the TME can inhibit DC migration. The occurrence of ectopic LNs in tumors can also induce DC migration via CCL21 (Chen et al., 2002; Di Caro et al., 2014). NK cells promote cDC1 accumulation in incipient tumors by producing CCL5 and XCL1/2 (Bottcher et al., 2018). Immunoregulatory factor PEG2 can downregulate the expression of chemokines CCL5, XCL1, XCR1, and CCR5 on cDC1 to inhibit DC accumulation and CD8^+^ T cell action in the TME (Bottcher et al., 2018). Regulating the expression of chemokines and/or chemokine receptors may interfere with the accumulation of DC in tumors or tumor-draining LNs.

A nano-vaccine containing M-COSA/OVA/pDNA can promote the expression of MHC-I and cytokines (such as IFN-γ) after injection into the human body, enhance antigen presentation as an immune adjuvant, induce DC migration to LNs, and activate CD8^+^ T-cell effects to inhibit tumor growth (Xiqin et al., 2018). The key to effective DC vaccines involves the migration of DC-carrying antigens to T-cell-rich LN regions. The use of magnetic resonance imaging, fluorescent labeling, and other technical methods to track the migration route of DC in the body enables DC vaccines to target their effects on cancer (de Chickera et al., 2011). In-depth understanding of DC migration routes are conducive to the preparation of targeted DC vaccines.

### Inflammation Cytokines

Inflammatory cytokines promote DC migration through paracrine or autocrine signaling and induce the expression of CCR7 and its ligand CCL19/CCL21 in DCs, thereby promoting DC migration (MartIn-Fontecha et al., 2003; Del Prete et al., 2006). The TNF-α, IL-6, and IL-1β families are involved in DC migration to inflammation sites that are under mediation by CCR7, and this process may be related to the Toll-like receptor/transcription factor nuclear factor-κB (TLR/NF-κB) pathway, which can modulate the Th1/Th17 polarization effect (Cumberbatch et al., 2002; Gianello et al., 2019). In addition, CX3CL1 and CXCL12 may participate in DC migration in the inflammatory environment and promote DC migration through the vascular endothelium to lymphatic vessels (Johnson and Jackson, 2013). In the non-inflammatory environment, the atypical Iκβ-dependent pathway activated by NF-κB appears to regulate CCR7 and co-stimulatory molecule expression (Baratin et al., 2015). At the same time, TLR ligands can enhance DCs to express CCR7 and CCL19 and promote DC migration from peripheral tissues to draining LNs (González et al., 2014). Thus, the inflammatory environment or inflammatory signals (TGF-α, IL-1β, IL-6, and IL-12) promote DC maturation and migration depending on CCR7 expression.

### Pathogenic Microbes

Invasion by pathogenic microbes affects the migration and location of DC subgroups. In the gut, CD103^+^CD11b^+^ cDC2 in the intestinal lamina propria of *Salmonella* infection upregulate CCR7 and migrate to the IEC layer, which helps epithelial DCs acquire bacterial infections (Farache et al., 2013). A substantial number of CCR2-dependent LY6C^*hi*^ monocytes that secrete proinflammatory factors and accumulate in the intestine may transform into inf-DCs and then migrate to mesenteric LNs and induce T-cell effects (Zigmond et al., 2012). However, acute intestinal bacterial infection may cause a substantial number of migrating DCs to converge in the adipose tissue area of mesenteric LNs, thereby preventing transfer to mesenteric LNs (Fonseca et al., 2015).

Respiratory syncytial virus (RSA) infection promoted CD11b^+^ DCs to carry allergens to mediastinal LNs by CCR2/CCL2 and CCR7, which induced Th2 cell immunity and caused allergic asthma (Plantinga et al., 2013). During RSV infection, the G protein receptor EOS1 caused the lung CD103^+^CD11b^+^ DC subgroup to migrate to mediastinal LNs (Lukens et al., 2009), thereby up-regulating the expressions of IL-18, IL-1, and IL-1R, which promoted respiratory DC migration and increased the inflammatory response (Pang et al., 2013). NDV-VLPs, an emerging virus vaccine (Qu et al., 2005), up-regulates MHC-II, co-stimulatory molecules, and proinflammatory cytokines TNF-α, IFN-γ, IL-6, and IL-12p70 through the TLR4/NF-κB pathway, thereby effectively activating DC maturity. In addition, NDV-VLPs induce the expression of CCR7 on DCs and cooperate with CCL19/CCL21 to mediate the migration of DCs to draining LNs or the spleen to activate CD4^+^ T-cell response. These discoveries provides new insight toward the development of similar VLP vaccines.

Notably, the coronavirus disease 2019 (COVID-19) infection also produces a large number of chemokines (CCL2, CCL3, CCL5, CXCL8, CXCL9, CXCL10, etc.), that might promote DCs and/or T cell infiltration into infected sites, thereby causing cytokine storms that destroy lung function and cause a systemic inflammatory response that leads to organ failure (Huang et al., 2020; Rivellese and Prediletto, 2020; Xu et al., 2020). Recently, it was found that inflammatory disease-inflammatory type 2 cDCs (inf-cDC2s) (Bosteels et al., 2020), which are structurally similar to DCs but have the combined functional advantages of monocytes, macrophages, and cDC functionality, exert an anti-inflammatory effect in COVID-19 patients.

### Others

Proteomic and transcriptome analyses confirmed that the *lnc-Dpf3* gene can negatively inhibit CCR7-mediated HIF-1α activation and glycolysis gene *LDHA* expression, ultimately negatively regulating CCR7-mediated DC migration and inflammation (Liu et al., 2019). This study shows that deletion of *lnc-Dpf3* gene can enhance CCR7-mediated activation of HIF-1α and DC migration and provides direction for research on the expression or role of non-coding long-chain RNAs in DC migration and inflammatory diseases.

In addition, laser irradiation or radiation may damage collagen fibers and the cell matrix of cells, thus causing collagen fibers to become disordered or broken, which affects the local recruitment of DC and promotes the migration of DCs to LNs (Chen et al., 2012; Yu et al., 2018). Laser-irradiated DCs may be accompanied by an increase in MHC-I and CD80 (Chen et al., 2012). It was established that, depending on the ATM/NF-κB signaling pathway, low-dose radiation may increase CCR7-mediated DC migration and is accompanied by an increased secretion of IL-12. Whether external infection factors or internal genetic factors affect DC migration by regulating the expression of chemotactic or adhesion factors in DCs (Table 2), further study of the mechanisms regulating the migration of DCs will elucidate important factors underlying the pathogenesis and immune status of disease.

**TABLE 2:** Factors affecting DCs migration.

Influence factors			Chemokines/chemokines receptor	Migration route	Immunological functions	References
Inhibition	Tumor microenvironment	TGF-β, VEGF, LXR ligands, anti-inflammatory factors or PGE2	CCR7-CCL19/CCL21; CCR5 and XCR1/XCL1	Inhibiting DCs to migrate from the tumor environment to the T cell cortex in tumor-draining lymph nodes.	Inhibition of CD8^+^ T-cell response.	Soudja et al. (2011); Bottcher et al. (2018)
	Hypoxia		CCR7-CCL19/CCL21 and adenosine receptor A2b	Inhibiting DCs to migrate toward draining lymph nodes (dLNs).	Imbalance of Th1/Th2 immune response.	Qu et al. (2005), Liu et al. (2019)
	Others	lnc-Dpf3 gene	CCR7	Inhibiting late-stage of DCs migration toward dLNs.	Inhibition of inflammation responses.	Liu et al. (2019)
Promotion	Inflammatory cytokines	TNF-α, IL-6, and IL-1β family	CCR7-CCL19/CCL21; CX3CL1 and CXCL12	Promoting DCs to migrate from peripheral tissues to dLNs.	Regulation the Th1/Th17 response.	Cumberbatch et al. (2002); Johnson and Jackson (2013), Gianello et al. (2019)
	Laser or Radiation	By up-regulate IL-12 or MHC-I, CD80	CCR7	Promoting DCs to migrate toward dLNs.	Damage the collagen fibers and cell matrix.	Chen et al. (2012), Yu et al. (2018)
	Vaccines (M-COSA/OVA/pDNA vaccine/NDV-VLPs vaccine)	By up-regulateTNF-α, IFN-γ, IL-6, IL-12p70 or MHC-I, IFN-γ	CCR7-CCL19/CCL21	Promoting DCs to migrate toward dLNs or spleen.	Activation of CD4^+^/8^+^ T response.	Qu et al. (2005); Xiqin et al. (2018)

## Conclusion and Outlook

The essence of DC migration involves chemokines, adhesion factors, integrins, and contributing biological activities. Different migration modes eventually lead to differences in the DC phenotype, location, and function. DC migration has a guiding role in the development and functions of DC-tumor or DC-virus vaccines, which are injected and migrated through blood vessels or lymphatic vessels, and eventually to the site of infection or tumor to play a role in antiviral or anti-tumorigenic effects (Figure 1B). Further studies are needed to determine whether the DC-tumor vaccine can effectively reach the local tumor to induce an anti-tumorigenic effect and whether DC-virus vaccine can effectively reach the infected site and elicit antiviral response. The development of transcriptomes, proteomics, and other technologies will provide technical support for more precise expression and regulation of DC migration to achieve a more effective treatment.

Migration from non-lymphoid to lymphoid tissue is a key feature of DCs that regulates immune response. Chemokine/chemokine receptors, integrins, protein receptors, and transcription factors, promote DC migration and specific intra-organ localization. In tumor tissues, removing inhibitory factors on DC migration may activate immunity and anti-tumorgenicity. However, inhibiting DC migration may be related to the inhibition of excessive activation in autoimmune diseases. Targeting CCR7 or other key mediators of DC trafficking may represent more suitable approaches for targeting DCs in diseases. Research on the migration and function of specific DC subgroups in diseases requires further study.

At present, research on genomes that affect DC migration and the migration modes of pDC, cDC, and other DC subgroups are unclear, and there is still a question about how to precisely target the direction of DC migration to make DC vaccines effective in antitumor and antiviral therapies. For the development and clinical application of an effective DC antiviral vaccine for COVID-19, which has rapidly spread around the world since December 2019, an in-depth exploration of changes in DC migration during the immune response to infection by pathogenic microorganisms is key, but greater elucidation is urgently needed.

## Author Contributions

WL and MF contributed toward the concept and manuscript writing. SZ, YY, QS, and XL participated in the literature search and discussion. All authors contributed to the article and approved the submitted version.

## Conflict of Interest

The authors declare that the research was conducted in the absence of any commercial or financial relationships that could be construed as a potential conflict of interest.
